# Progress and barriers to the implementation of prediction modelling in child and adolescent mental health—A commentary on Senior et al. (2021)

**DOI:** 10.1002/jcv2.12052

**Published:** 2021-11-22

**Authors:** Alan J. Meehan, Andrea Danese

**Affiliations:** ^1^ Social, Genetic & Developmental Psychiatry Centre Institute of Psychiatry, Psychology & Neuroscience King’s College London London UK; ^2^ Yale Child Study Center Yale School of Medicine New Haven Connecticut USA; ^3^ Department of Child & Adolescent Psychiatry Institute of Psychiatry, Psychology & Neuroscience King’s College London London UK; ^4^ National and Specialist CAMHS Trauma, Anxiety, and Depression Clinic South London and Maudsley NHS Foundation Trust London UK

## INTRODUCTION

As clinicians seek to enhance detection, prognosis and treatment planning for young people with mental health difficulties, the benefits in moving towards more individualised risk prediction has become increasingly apparent. Although epidemiological evidence has historically identified important factors whose presence confers an increased average risk for a given mental health outcome between defined population sub‐groups, such risk factors may not accurately predict an individual’s specific likelihood of exhibiting that outcome. More recently, the application of bespoke statistical models that account for individual variation in risk has supported successful development and integration of structured prediction tools into routine practice (e.g. diagnostic screening, risk assessment and resource allocation) across clinical medicine. Progress towards a personalised medicine infrastructure for mental health has been slower than other areas of medicine, but a growing number of systematic reviews have mapped the recent consolidation of the field of ‘precision psychiatry’ within clinical research (e.g. Salazar de Pablo et al., 2021).

This conceptual shift towards individualised risk prediction has required methodological innovation. On a technical level, the complexity of the statistical techniques underlying psychiatric prediction models ranges from relatively straightforward extensions of the conventional regression framework to unsupervised machine‐learning techniques that can generate predictions using large pools of candidate predictors with limited pre‐specification. However, as the field has progressed, consistent methodological standards have also emerged. In particular, predictive performance should be directly tested using metrics of individual classification – specifically, discrimination and calibration. The datasets used to develop prediction models should be large enough to minimise inflation of apparent predictive ability due to sample‐specific features or idiosyncrasies, known as ‘overfitting’. Moreover, to ensure reliable predictions in new individuals (or ‘generalisability’), prediction models need to be extensively validated in unused cases from the original development dataset (internal validation) and, ideally, new datasets (external validation). Finally, for translation into clinical practice to be successful, even the most robustly validated prediction models must balance the competing risks of under‐detection (i.e. missed ‘at‐risk’ patients) and over‐detection (i.e. inaccurate classification of ‘low‐risk’ patients) depending on the specific clinical decision at hand (Fusar‐Poli et al., 2018).

With consensus around the quality and reporting of prediction models increasingly codified in formal best practice guidelines (e.g. Wolff et al., 2019), the recent systematic review by Senior et al. (2021) represents the first published synthesis of methodological progress towards psychiatric prediction models specifically for child and adolescent populations (i.e. aged ≤18 years). This developmental period is particularly relevant to efforts to predict the onset of mental health conditions, given well‐established evidence that up to 75% of psychiatric disorders emerge by the age of 18 (Kessler et al., 2007). The authors identified 100 eligible publications from the past 3 years alone, highlighting the rapid growth of this field. However, to ensure that methodological innovation leads to clinical improvement, it is important that the exponential rise in published prediction models does not come at the prize of inadequate analytic quality and, in turn, poor clinical utility. We will next discuss these challenges in relation to the evidence base summarised in this review, as well as their broader implications for child and adolescent mental health.

## ANALYTIC QUALITY

One of the most striking findings reported by Senior et al. (2021) is that only 2 of the 100 eligible studies demonstrated a low ‘risk of bias’, driven both by inappropriate analytic strategies and insufficient reporting of key study characteristics. Risk of bias was evaluated across four domains (participants, predictors, outcome and analysis) using the Prediction model Risk Of Bias ASsessment Tool (PROBAST; Wolff et al., 2019). This checklist utilises a ‘worst score counts’ framework, whereby any study feature deemed to confer a high risk of bias results in that domain receiving a ‘high risk’ classification; in turn, if any one domain is deemed ‘high‐risk’, this label is applied to the entire study. This relatively strict scoring system necessitates examining the individual PROBAST components more closely.

Across the reviewed literature, the most common source of bias was the ‘analysis’ domain. Returning to the methodological requirements outlined above, most included studies provided incomplete evaluations of their models’ predictive performance. Specifically, although 75% presented performance metrics for discrimination (the ability to accurately differentiate those with and without the outcome), only 26% reported the equally important measure of calibration (the overall agreement between predicted and observed values). Low statistical power was also persistent: only one‐third of model development studies met the threshold of at least 10 ‘events per variable’ (EPV), putting them at increased risk of overfitting. This metric’s emphasis on the number of outcome events in a dataset, rather than overall sample size, is particularly relevant for studies attempting to predict relatively rare outcomes (e.g. suicide). Although debates around sufficient EPV benchmarks are ongoing, most statistical research recommends a higher EPV ratio of at least 20 (Ogundimu et al., 2016). If applied, only 10 of the 52 development studies in this review would meet this more stringent criterion.

In terms of model generalisability, only six studies presented evidence of external validation in an independent dataset. For the remainder of studies, the extent to which reported predictive performance in the initial development sample can be reliably extrapolated to new individuals therefore remains unknown. Results also suggest that external validation is not a panacea for overfitting, as the majority of validation samples did not include at least 100 events (individuals with the outcome), which has been routinely recommended to ensure adequate power (Fusar‐Poli et al., 2018). The authors’ suggestion that future protocols include plans for external validation would not only promote more robust external validation of prediction models before they are widely disseminated, but also ensure that proposed external datasets are of an adequate size.

On a more general note, it remains somewhat difficult to determine whether these prediction models are genuinely at high risk of bias, or if such ratings are simply the result of these key study characteristics and performance metrics being underreported. Despite their focus on ‘current models’ published since 2018, Senior et al. (2021) acknowledge that standardised guidelines for reporting prediction studies have only recently been disseminated, and it may take some time for them to be applied more consistently across empirical research. These efforts may be aided by adherence to reporting tools, such as the Transparent Reporting of a multivariate prediction model for Individual Prognosis or Diagnosis (TRIPOD) checklist (Collins et al., 2015), and an expectation from academic journals that submitted prediction modelling manuscripts include a completed PROBAST assessment.

## CLINICAL UTILITY

Although Senior et al.’s (2021) recommendations primarily focus on aspects of methodology and reporting, none of the prediction tools they reviewed could be recommended for use in clinical practice. This exemplifies the wider point that even well‐performing and independently validated models need to demonstrate meaningful translation potential before clinical application can realistically be considered. Among the barriers to implementation identified in the review, the degree to which model development samples were representative of their intended clinical population warrants particular attention. Once again, incomplete reporting of sample characteristics impeded the authors' ability to reliably evaluate clinical applicability. Reliance on sample selection, either through case‐control designs or by specifying subsamples within community‐based cohorts, may also yield biased datasets and optimistic estimates of predictive performance.

One interesting suggestion by the authors is that models developed within specific at‐risk groups (child protection [e.g. Meehan et al., 2020], juvenile justice settings) may provide more accurate and clinically useful predictions than universal approaches seeking to predict onset of mental illness across the general population. This approach to sample selection may generate models that, though less universally applicable, may more successfully close the clear ‘translation gap’ for prediction models within child and adolescent mental health. One promising avenue to expand this ‘at‐risk’ approach is the use of electronic health records, which, by providing a contemporaneous account of a patient’s progress through psychiatric care, could offer a basis for prediction models based solely on information that is routinely collected by clinicians during assessment and treatment. In contrast, notwithstanding some notable exceptions highlighted by the authors (e.g. Rocha et al., 2021), efforts to examine psychiatric prediction from a global health perspective are still in their infancy. Due to limited cross‐country validation of existing prediction models, relative underdevelopment of new models in lower‐ and middle‐income countries, and wide global variations in healthcare systems, the goal of a universal prediction model within child and adolescent mental health may be too ambitious.

Only three model development studies based their predictor configuration on expert opinion or stakeholder input, with the remainder employing some data‐driven method of predictor selection that risked limiting their applicability in real‐world settings. Current methodological guidelines generally recommend *a priori* predictor selection informed by clinical knowledge, both to minimise the potential for biased coefficients and to ensure that the proposed model sufficiently resembles the clinical context for which it is intended. Recent empirical evidence has also suggested that such a ‘clinical learning’ approach can yield levels of predictive performance comparable to machine‐learning methods which, although more sophisticated, may introduce additional implementation challenges (Fusar‐Poli et al., 2019).

An important limiting characteristic of the literature search was the exclusion of models whose predictors were comprised solely of neuroimaging or genetic data. The authors’ assertion that effective clinical translation of these models remains rare aligns with recent reviews that have discussed the challenges of implementing neuroimaging and genetic prediction studies within psychiatry (Bracher‐Smith et al., 2021; Rashid & Calhoun, 2020). In brief, from a methodological perspective, these studies have typically relied on case‐control designs with a relatively small number of outcome events relative to a large number of predictive features extracted from high‐dimensional datasets. Furthermore, on a more pragmatic note, the complexity and cost involved in collecting and processing these data at present is likely to make their use within routine clinical settings impractical, and further underscores the need to develop prediction models based on readily available data to fully maximise their potential utility.

Finally, while the characteristics of a given prediction model can be used to make inferences around its relevance to real‐world practice, the authors rightfully acknowledge that there are specific criteria available to evaluate clinical utility. Key concepts for consideration include the feasibility of adapting the model for use within clinical workflows (i.e. availability of predictors, interpretability of output and overall cost‐effectiveness) and its overall acceptability – both to the clinicians expected to use it and the young people who will ultimately be affected by its decisions (Fusar‐Poli et al., 2018). Although statistical methods have been devised to formally quantify the likely benefit of implementation compared to current screening or assessment protocols, it is also crucial to engage with relevant stakeholders (e.g. patients, parents and clinicians) from the outset of model development to identify potential challenges and barriers. Of particular relevance to child and adolescent settings are issues around the potential stigma of being labelled as ‘at‐risk’ for a given mental health outcome, and the extent to which that label will be used to inform appropriate preventive interventions.

## CONCLUSION

Despite the rapid proliferation of published prediction models for child and adolescent mental health in recent years, individual risk prediction is not yet ready for implementation within routine clinical settings. To ensure that this methodological progress leads to tangible improvements in psychiatric screening and assessment, it is crucial that the current enthusiasm for generating novel prediction models for psychiatric outcomes does not come at the expense of adequate quality, thorough reporting and clinical utility. By both summarising progress made in the field to date and highlighting key challenges still to be addressed, Senior et al.’s (2021) systematic review provides us with a promising glimpse of the potential improvements this individualised approach could bring to clinical practice, along with a potential roadmap on how we can get there.

## CONFLICT OF INTEREST

The authors have declared that they have no competing or potential conflicts of interest.

## Data Availability

Data sharing not applicable to this article as no datasets were generated or analyzed during the current study.
